# Assessment of long COVID symptom burden in patients testing positive for SARS-CoV-2 at a nationwide retail pharmacy

**DOI:** 10.1371/journal.pone.0345639

**Published:** 2026-03-25

**Authors:** Xiaowu Sun, Joseph C. Cappelleri, Laura L. Lupton, Mary M. Moran, Santiago M. C. Lopez, Laura Puzniak, Alon Yehoshua, Manuela Di Fusco

**Affiliations:** 1 Life Sciences Solutions, CVS Healthspire^TM^, CVS Health®, Wellesley, Massachusetts, United States of America; 2 Statistical Research and Data Science Center, Pfizer Inc., Groton, Connecticut, United States of America; 3 MDSCA Vaccines, Pfizer Inc., Collegeville, Pennsylvania, United States of America; 4 Health Economics and Outcomes Research, Pfizer Inc., New York, New York, United States of America; Northwestern University Feinberg School of Medicine, UNITED STATES OF AMERICA

## Abstract

**Background:**

Numerous grouping and scoring methodologies have been proposed to assess long COVID symptomatology. One approach is to use symptom count as a simple, quantifiable measure of long COVID symptom burden.

**Methods:**

This was a secondary analysis of a nationwide patient-reported outcomes (PRO) study that recruited symptomatic adults testing positive for SARS-CoV-2 at a Retail Pharmacy in the spring of 2023. EQ-5D-5L^TM^, work productivity and impairment (WPAI), the Patient-Reported Outcomes Measurement Information System (PROMIS^®^) fatigue, and a long COVID symptom questionnaire were administered at Week 4, Month 3, and Month 6 after testing. Pre-infection EQ-5D-5L, WPAI, PROMIS fatigue were collected via recall. Cronbach’s α assessed internal consistency of symptoms. Scree plots determined number of significant factors (symptoms) to retain for analysis. Spearman correlation coefficients were calculated between number of symptoms and EQ-5D-5L, WPAI, PROMIS fatigue scores and their changes from pre-COVID baseline. Categorization of long COVID burden using number of symptoms was proposed based on scores via equipercentile linking.

**Results:**

Of 505 patients, mean age was 46.3 years, 70.7% were female. Cronbach’s α was 0.865, denoting good internal consistency of the symptom survey instrument. The scree plot supported use of one factor for the composite 30-symptom list. Number of symptoms correlated strongly with EuroQol Utility Index (r = −0.53), presenteeism (r = 0.51), activity impairment (r = 0.51) and fatigue (r = 0.56). Statistically significant differences in mean number of symptoms were found between patients with versus those without problems in any of the 5 domains of the EQ-5-dimensional descriptive system. Based on linked PRO scores, subjects could be classified into low (≤2), medium (3–9), and high (≥10) symptom burden.

**Conclusions:**

Number of long COVID symptoms correlated with validated PRO measures and identified three symptom-based categories of long COVID burden. Number of symptoms is a valid and internally consistent measure to assess long COVID burden in outpatient settings.

## Background

The US Centers for Disease Control and Prevention (CDC) refers to long COVID as signs, symptoms and conditions that persist or develop following a SARS-CoV-2 infection [1–3]. To date, several leading organizations—including the WHO, the CDC, and the National Institute for Health and Care Excellence—have proposed definitions, though important differences remain among them [1,4–6]. Consequently, long COVID still lacks a harmonized definition. Moreover, as of today, there is currently no approved treatment [3]. The existing body of evidence suggests that long COVID represents more than one syndrome, manifesting in variable disease patterns with a wide range of over 200 symptoms [7] affecting multiple organ systems [2]. Studies have shown that long COVID has great individual variability in the type and severity of symptoms and is influenced by factors such as age, overall health, and severity of the initial infection [8]. Causal factors may include persistent viral reservoirs, immune system dysfunction, and microvascular damage [2].

The Household Pulse Survey (HPS) conducted between August 20 and September 16 2024 found that 8.7% of those reporting a current or prior SARS-CoV-2 infection (5.3% of all adults in the United States) reported currently experiencing symptoms of long COVID [9], suggesting long COVID remains a public health concern.

Long COVID poses a substantial clinical, economic, and humanistic burden. Studies show that it may cause long-lasting physical, mental, and emotional impairments, reducing quality of life, activity levels, and the ability to work [10,11]. One study estimated that long COVID may have cost the United States about $2.6 trillion dollars through 2022 [12].

Given the multifactorial nature of long COVID, several methods have been explored in the literature to break down its complexity. Two distinct approaches have been applied to the assessment of long COVID: grouping by patients or grouping by symptoms. The former identifies long COVID subtypes that differ in symptom patterns and provides patient-level stratification and symptom phenotypes, but only indirect evidence on disease severity. Methods for grouping patients include cluster analysis, latent class analysis, and machine learning to identify subtypes of long COVID [13–16].The latter (grouping of symptoms) may advance the development of a scoring methodology [17–19] to estimate disease severity. For example, the symptom burden questionnaire for long COVID [17] is comprised of 17 subscales with 131 symptoms, and each subscale has score 0–100 which is converted from the summation of symptom ratings. In an outpatient setting, Ye et al. (2024) reported the development of a Long COVID Symptom and Severity Score (LC-SSS) in college students with SARS-CoV-2 infection [20]. LC-SSS was calculated by summing ratings of 44 symptoms, each from 0 to 3. Unidimensional structure was verified with a confirmatory factor analysis. Then mild, moderate, and severe clusters were proposed based on LC-SSS.

Patient based clustering is useful for defining symptom phenotypes, whereas symptom count scoring provides a simpler and scalable measure of overall symptom burden once its measurement properties are established. In an outpatient setting, however, the low/medium/high symptom phenotypes identified symptom phenotypes via latent class analysis [16] were subsequently mapped to simplified to number of symptom-based categories [21].

Using data from a previously published PRO study [22], this methodological study aims to further the understanding of long COVID by exploring whether number of symptoms can be used as a reliable measure for burden of disease. First, the study checked the dimensional structure and psychometric properties of a composite 30-symptom list, and sub-lists of 19-item CDC list [1] and 12-item HPS list [23]. Next, the relationship between symptoms and patient-reported well-being and work productivity was explored through equipercentile linking. Then, burden of disease categories were proposed using number of symptoms aligned to patient-reported outcomes (PRO) scores.

## Methods

### Data

A nationwide prospective patient-reported outcomes (PRO) survey study (clinicaltrials.gov NCT05160636) was conducted in 2023 [22]. Individuals aged 18 years and older who tested positive for SARS-CoV-2 at a U.S. national retail pharmacy chain and were symptomatic at the time of testing were invited via email to participate between 2 March and 18 May 2023, and were followed for 6 months post-infection. Long COVID symptoms were collected at Week 4, Month 3, and Month 6 after the positive test. Of the 643 patients surveyed, 505 patients completed the long COVID symptom questionnaire at one or more follow-up time points over the 6 month follow up period and were included in the analysis. The baseline characteristics of study participants, including demographics, comorbidities, living and work settings, and COVID-19 vaccination history were obtained via a pre-test screening questionnaire that patients completed upon registration for testing. Self-reported PRO measures of health-related quality of life (HRQoL), work productivity and activity levels were assessed using validated instruments (EQ-5D-5L^TM^, WPAI, PROMIS^®^ Fatigue 8a). All data were collected electronically.

### Long COVID symptoms

Long-COVID symptoms were self-reported via a 30-symptom questionnaire that was developed by the interdisciplinary study team based on long COVID symptoms available in the prevailing literature [24,25]. Specifically, the questionnaire expanded the symptoms in the long COVID symptoms list from the CDC [1]. Redundant symptoms bearing similar meanings or content were removed based on the clinical experience of the medical experts on the study team. The resulting questionnaire included 30 unique symptoms that encompassed a wide range of symptoms associated with long COVID. The symptoms were organized in 5 categories with support from the medical experts: general symptoms (N = 6), cardiac and respiratory symptoms (N = 5), neurologic symptoms (N = 11), digestive symptoms (N = 4), and other symptoms (N = 4). These categories of symptoms have been previously described [22].

Participants were asked to answer the following question about long COVID symptoms: ‘Do you experience any of the following Ongoing COVID-related symptoms or NEW symptoms TODAY that you did not have before your participation in this survey study? (check all that apply)’. Symptoms that were not selected by the patient were considered “no”. resulting in a binary response format.

See supplementary S1 Table for details of each list. The surveys were administered on Week 4, Month 3 and Month 6.

### Patient-reported outcome measures

#### EQ-5D-5L.

The EuroQol 5-dimensional 5-level descriptive system (EQ-5D-5L) questionnaire developed by the EuroQoL provides a generic measure of health-related quality of life (HRQoL) [26]. The EQ-5D-5L has two components: a 5-question descriptive system and one visual analog scale (VAS). The EQ-5D-5L descriptive system comprises five dimensions: mobility [MO], self-care [SC], usual activity [UA], pain/discomfort [PD], and anxiety/depression [AD]. Each dimension has five response levels which are no problems, mild problems, moderate problems, severe problems, and unable to or extreme. The VAS allows respondents to rate their current health on a 101-point scale ranging from 0 = “Worst imaginable health state” to 100 = “Best imaginable health state.” The reported health states of the five dimensions were converted into a utility index (UI) for the U.S. general population, which ranges from −0.573 to 1 [27].

#### WPAI:GH.

The Work Productivity and Activity Impairment Questionnaire: General Health V2.0 (WPAI:GH) is a 6-item PRO questionnaire designed to assess work productivity and ability to perform regular daily activities during the past 7 days, excluding “today” [28,29]. Four scores were computed: percentage of work time missed (absenteeism), percentage of impairment while working (presenteeism), percentage of overall work impairment (work productivity loss), and percentage of activity impairment. Higher scores represent lower productivity and more daily activity impairment.

#### PROMIS Fatigue 8a.

To measure fatigue in adults, the Patient-Reported Outcomes Measurement Information System (PROMIS) Fatigue 8a is a short-form fixed instrument composed of 8 items measuring the experience and impact of fatigue from the 90-item PROMIS-Fatigue item bank [30]. The recall period is the past 7 days. Five responses for each item are scored 1–5 so that the raw score of 8 items total ranges from 8 to 40. The raw score is then converted to standardized T-score. The T-score has a mean of 50 and standard deviation (SD) of 10 in a referent population [31]. Higher T-scores indicate more severe fatigue. T-score can be interpreted as ‘Within Normal Limits’ if <55, ‘Mild’ if ≥55 to <60, ‘Moderate’ if ≥60 to <70, or ‘Severe’ if ≥70 [31].

PROM surveys were conducted post testing at Day 3 (EQ-5D-5L) and Week 1 (WPAI:GH and PROMIS Fatigue 8a). The EQ-5D-5L, WPAI:GH, and PROMIS Fatigue 8a questionnaires were also administered at Week 2, Week 4, Month 3, and Month 6 post testing [22]. To evaluate patients’ status pre-COVID, questionnaires were modified with added text of “BEFORE you experienced COVID-19 symptoms”. The modified pre-COVID “recall” versions were administered only on Day 3 (EQ-5D-5L) and Week 1 (WPAI:GH and PROMIS Fatigue 8a). On these days, the standard versions of the same questionnaires were also administered.

### Statistical analysis

Descriptive statistics were used to summarize participant characteristics and long COVID symptoms. Mean and standard deviation were used for continuous variables, and frequency and percentages for categorical variables.

Internal consistency, Cronbach’s α, was calculated for long COVID symptoms. Values of ≥0.7 (but less than 0.8) and ≥0.8 are considered acceptable and good, respectively [32]. Polychoric correlation coefficients were calculated between pairs of long COVID symptoms. Then exploratory factor analysis was conducted. The scree plots were utilized to help determine number of factors to be used [33]. To evaluate criterion validity, Spearman correlation coefficients were calculated between number of long COVID symptoms and other PROM scores and changes from pre-COVID. Strength of correlation was considered weak if 0.10–0.29, moderate if 0.30–0.49, or strong if ≥0.5 [34]. To evaluate known-group validity, number of symptoms was summarized by the status of with problems (mild, moderate, severe, or unable to/extreme) and without problems on each EQ-5D-5L dimension. Wilcoxon rank-sum tests were used to test the significance [35], and (standardized) effect sizes (ES) in terms of Cohen’s d statistics were calculated between groups with and without problems. Values of 0.2, 0.5, and 0.8 standard deviation units represent “small”, “medium”, and “large” effect sizes, respectively [34].

Equipercentile linking aligns percentile ranks across instruments (e.g., EQ‑5D‑5L, Utility Index, VAS, WPAI, PROMIS Fatigue) to identify score values that are equivalent in their empirical distributions. This facilitates cross‑measure interpretation. Equipercentile linking is distribution‑agnostic and is appropriate for skewed and ordinal scales, making it well suited to the present data [36]. Linkages were arranged in table form for visualization of alignment between number of symptoms and other PRO measures. Linking number of symptoms to external validated anchors allowed us to propose categories of low, medium, and high symptom burden as proposed in other studies [16,20]. The final cut-points were selected to balance clinical interpretability and alignment with PRO distribution percentiles. Although EQ 5D 5L and WPAI do not have universally accepted clinical thresholds and PROMIS thresholds do not align directly with symptom counts, the equipercentile linking table provides a transparent mapping across instruments and symptom counts. This allows readers to evaluate the chosen cut points and to consider alternative thresholds or numbers of categories.

Analyses were performed using the 30-symptom list and then replicated with symptoms from the CDC list and HPS list. All the analyses used data pooled from the 3 follow-up time points (week 4, month 3, month 6). Some results are presented as supplemental material. Equipercentile linking was implemented using SAS macro EQUIPERCENT [37]. All analyses were conducted with SAS Version 9.4 (SAS Institute, Cary, NC).

### Ethics approval and consent to participate

This study was approved by the Sterling IRB, Protocol #C4591034. Participation in the study was voluntary. Written consent was obtained electronically. Participants were informed of their right to refuse or withdraw from the study at any time. Participants were compensated for their time.

## Results

### Study participants

The cohort was previously described [22]. In these analyses, 505 (100%), 470 (93%), and 444 (88%) patients completed the Week 4, Month 3, and Month 6 surveys for long COVID symptoms, respectively. The average age of participants was 46.3 (SD: 15.5) years, 70.7% were female, 60.4% were White, 25.1% had ≥ 1 comorbidity, and 40.4% had previously tested positive for SARS-CoV-2 [22].

### Long COVID symptoms and PROs

As previously reported, the top 3 most frequently reported symptoms were fatigue, brain fog, and cough at Week 4, and fatigue, brain fog, and sleep problems at Month 3 and Month 6 [22]. From Week 4 to Month 6, patients on average experienced a slightly declining but similar number of symptoms. At Month 6, the mean (SD) numbers of long COVID symptoms for the composite 30-symptom, CDC, and HPS lists were 2.2 (2.6), 1.2 (2.1), and 0.9 (1.6), respectively. Compared with pre-COVID, higher percentages of patients reported problems in all EQ-5D-5L dimensions from Week 4 to Month 6 after infection. Numerically, there was an improving trend in mobility and usual activity and a worsening trend in the pain/discomfort and anxiety/depression dimensions from Week 4 to Month 6. (Table 1)

**Table 1:** Summary of Number of Long COVID Symptoms and Patient-Reported Outcome Measures.

	Pre-COVID (N = 643)	Time point			
		Pooled (N = 1,407)	Week 4 (N = 505)	Month 3 (N = 470)	Month 6 (N = 444)
Number of symptoms, mean (SD)					
30-symptom list	–	2.4 (3.0)	2.6 (3.1)	2.4 (3.1)	2.2 (2.6)
CDC list	–	1.4 (2.3)	1.7 (2.3)	1.4 (2.4)	1.2 (2.1)
HPS list	–	1.0 (1.7)	1.2 (1.8)	1.0 (1.7)	0.9 (1.6)
EQ-5D-5L					
VAS score, mean (SD)	86.0 (12.0)	85.3 (11.6)	85.3 (11.5)	85.9 (10.9)	84.8 (12.6)
Utility Index (US) score, mean (SD)	0.93 (0.11)	0.90 (0.14)	0.90 (0.14)	0.91 (0.14)	0.90 (0.14)
Problem in each Dimension, n (%)					
Mobility	39 (6.1)	179 (12.6)	70 (13.8)	56 (11.9)	53 (11.9)
Self-care	14 (2.2)	72 (5.1)	26 (5.1)	21 (4.5)	25 (5.6)
Usual activities	59 (9.2)	301 (21.2)	130 (25.7)	91 (19.3)	80 (17.9)
Pain/ Discomfort	177 (27.5)	515 (36.2)	182 (36.0)	163 (34.6)	170 (38.1)
Anxiety/ Depression	263 (40.9)	612 (43.0)	207 (40.9)	204 (43.3)	201 (45.1)
WPAI:GH score, mean (SD)					
Absenteeism	10.4 (24.3)	6.7 (16.8)	6.2 (15.7)	5.9 (15.8)	8.1 (18.9)
Presenteeism	14.0 (23.7)	13.7 (20.7)	15.0 (21.6)	13.0 (19.5)	13.0 (20.7)
Work Productivity Loss	18.4 (27.6)	18.3 (24.6)	19.1 (25.5)	17.2 (23.4)	18.4 (24.9)
Activity Impairment	16.4 (25.1)	15.6 (22.3)	16.9 (22.5)	15.2 (22.8)	14.4 (21.7)
PROMIS Fatigue 8aT-score, mean (SD)	44.7 (9.5)	47.7 (10.1)	47.6 (10.2)	47.6 (10.2)	47.9 (9.9)

Abbreviations: CDC = Centers for Disease Control and Prevention; EQ-5D-5L = 5-level version of the EuroQol 5-dimensional descriptive system; HPS = Household Pulse Survey; PROMIS = Patient-Reported Outcomes Measurement Information System; SD = standard deviation; US = United States; VAS = visual analog scale; WPAI:GH = Work Productivity and Activity Impairment Questionnaire: General Health

### Questionnaire validation

**Fig 1:**
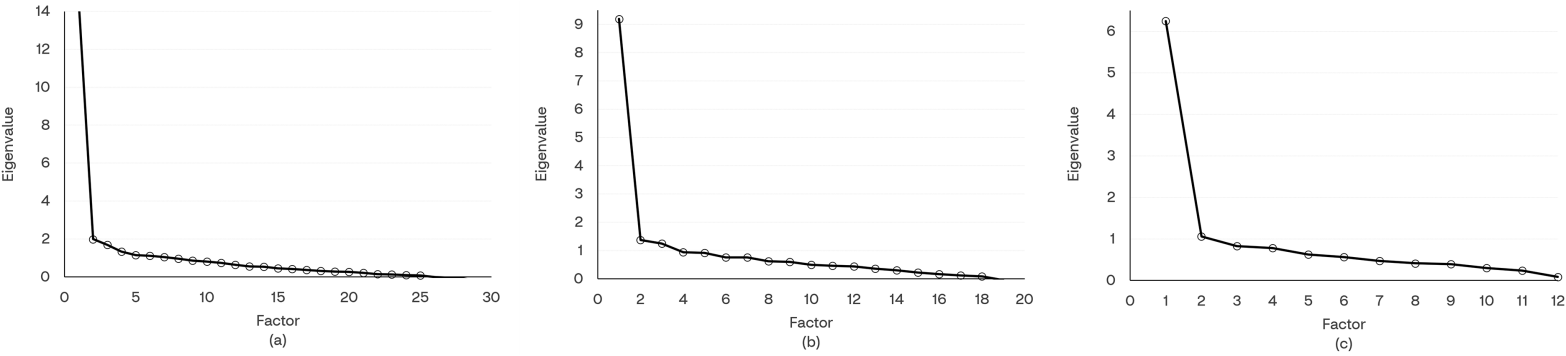
Scree Plots for Long COVID Symptoms. **(a)** All symptoms **(b)** CDC list **(c)** HPS list. Abbreviations: CDC = Centers for Disease Control and Prevention; HPS = Household Pulse Survey.

The internal consistency with Cronbach’s α, the scree plot for the exploratory factor analysis, Spearman correlations for criterion validity, and results for known-group validity are presented in Tables 2–4 and Fig 1, along with supplementary data presented in S1–S4 Tables.

**Table 2:** Spearman Correlations Between Number of Symptoms and Patient-Reported Outcome Measures.

	30-symptom list	CDC list	HPS list
EQ-5D-5L			
VAS	−0.37	−0.36	−0.35
UI	−0.53	−0.53	−0.51
WPAI:GH			
Absenteeism	0.19	0.17	0.15
Presenteeism	0.51	0.50	0.49
Work Productivity Loss	0.44	0.42	0.41
Activity Impairment	0.51	0.52	0.51
PROMIS Fatigue 8a T-score	0.56	0.57	0.57

All *P* values <0.001.

Abbreviations: CDC = Centers for Disease Control and Prevention; EQ-5D-5L = 5-level version of the EuroQol 5-dimensional descriptive system; HPS = Household Pulse Survey; PROMIS = Patient-Reported Outcomes Measurement Information System; UI = Utility Index; VAS = visual analog scale; WPAI:GH = Work Productivity and Activity Impairment Questionnaire: General Health

**Table 3:** Spearman Correlations Between Number of Symptoms and Patient-Reported Outcome Measures Change from Pre-COVID.

	30-symptom list	CDC list	HPS list
EQ-5D-5L			
VAS	−0.29	−0.28	−0.27
UI	−0.41	−0.41	−0.38
WPAI:GH			
Absenteeism	0.17	0.15	0.14
Presenteeism	0.31	0.30	0.31
Work Productivity Loss	0.31	0.28	0.28
Activity Impairment	0.25	0.24	0.24
PROMIS Fatigue 8a T-score	0.36	0.37	0.37

All *P* values <0.001.

Abbreviations: CDC = Centers for Disease Control and Prevention; EQ-5D-5L = 5-level version of the EuroQol 5-dimensional descriptive system; HPS = Household Pulse Survey; PROMIS = Patient-Reported Outcomes Measurement Information System; UI = Utility Index; VAS = visual analog scale; WPAI:GH = Work Productivity and Activity Impairment Questionnaire: General Health

**Table 4:** Comparing Number of Symptoms Between Patients With and Without Problem in EQ-5D-5L Dimensions: Mean (SD).

EQ-5D-5L dimension	30-symptom list			CDC list			HPS list		
	No problem	With problem	ES	No problem	With problem	ES	No problem	With problem	ES
Mobility	2.1 (2.5)	4.8 (4.7)	0.72	1.2 (2.0)	3.2 (3.4)	0.75	0.8 (1.5)	2.4 (2.6)	0.77
Self-Care	2.2 (2.7)	5.8 (5.2)	0.87	1.3 (2.2)	3.8 (3.5)	0.86	0.9 (1.6)	2.8 (2.6)	0.88
Usual Activities	1.7 (1.9)	5.1 (4.5)	1.00	0.8 (1.5)	3.6 (3.2)	1.12	0.6 (1.1)	2.7 (2.4)	1.15
Pain/ Discomfort	1.6 (1.7)	3.9 (4.0)	0.76	0.7 (1.4)	2.6 (3.0)	0.82	0.5 (1.1)	1.9 (2.2)	0.79
Anxiety/ Depression	1.5 (1.4)	3.6 (4.0)	0.69	0.7 (1.3)	2.4 (2.9)	0.76	0.5 (1.0)	1.7 (2.2)	0.73

All *P* values of Wilcoxon test comparing number of symptoms between patients with and without problem <0.001.

Abbreviations: CDC = Centers for Disease Control and Prevention; EQ-5D-5L = 5-level version of the EuroQol 5-dimensional descriptive system; ES = effect size; HPS = Household Pulse Survey; SD = standard deviation

Internal consistency Cronbach’s α for the composite 30-symptom list, CDC list, and HPS list were 0.87, 0.81, and 0.76, respectively. The ranges of Cronbach’s α corresponding to a symptom list after deleting each individual symptom were 0.86–0.87, 0.79–0.81, and 0.73–0.76 for the composite 30-symptom, CDC, and HPS lists, respectively. Therefore, the internal consistency was good for the 30-item composite list, between acceptable and good for the 19-item CDC list, and acceptable for the 12-item HPS list (S1 Table).

Scree plots were used to determine the number of factors to retain for analysis, and the plots suggested one-factor structure was appropriate for all 3 lists of long COVID symptoms. (Fig 1)

Number of symptoms correlated strongly with EQ-UI (r = −0.53), presenteeism (r = 0.51), activity impairment (r = 0.51), and fatigue (r = 0.56); moderately with EQ-VAS (r = −0.37) and work productivity loss (r = 0.44), and weakly with absenteeism (r = 0.19). (Table 2) Number of symptoms with changes from pre-COVID correlated moderately in EQ-UI (r = −0.41), presenteeism (r = 0.31), work productivity loss (r = 0.31), and fatigue (r = 0.36), and weakly in EQ-VAS (r = −0.29), absenteeism (r = 0.17), and activity impairment (r = 0.25). (Table 3) Similar strengths of association were found with the CDC and HPS lists, as shown in Table 3.

Differences in mean number of symptoms varied from 2.1 between patients with and without problems in anxiety/depression to 3.6 between patients with and without problems in self-care. All differences with respect to 5 dimensions were statistically significant (P < 0.001). Standardized effect sizes were all medium or large, and the largest effect size was found for usual activities. Similar results were also found for the CDC and HPS lists. (Table 4)

### Interpretation of number of symptoms with linked outcomes

Each of the 5 EQ-5D-5L domains, UI, VAS, WPAI scores, and PROMIS Fatigue score was aligned to number of symptoms via equipercentile linking. Presenting these linkages in table form provided visualization of number of symptoms aligned to each of the other PRO measures. Results for the 30-symptom list are presented in Table 5. Similar linked tables for the CDC and HPS lists are presented in S2 and S3 Tables.

**Table 5:** Number of Symptoms Linked to Patient-Reported Outcome Measures.

Number of Symptoms		PROMIS Fatigue T-score	EQ-5D-5L Dimensions^a^					EQ-5D-5L Scores		WPAI: GH Scores^d^			
n	%		AD	PD	UA	MO	SC	UI (US)^b^	VAS^c^	Activity Impairment	Work Productivity Loss	Presenteeism	Absenteeism
1	65.4	44	1	1	1	1	1	1	91	0	0	0	0
2	9.3	53	2	2	1	1	1	0.88	81	20	20	20	0
3	6.62	56	2	2	1	1	1	0.84	80	30	30	20	0
4	4.44	58	2	2	2	1	1	0.81	75	40	49	30	5
5	3.31	59	2	2	2	2	1	0.75	71	50	50	30	20
6	2.18	61	3	2	2	2	1	0.72	70	50	60	40	32
7	2.47	63	3	2	2	2	1	0.66	69	60	65	60	50
8	1.48	64	3	3	2	2	2	0.62	65	70	70	60	50
9	0.78	65	3	3	2	2	2	0.58	61	70	75	70	50
10	0.85	66	3	3	3	2	2	0.56	60	80	78	70	50
11	0.56	66	4	3	3	2	2	0.53	56	80	80	70	50
12	0.78	67	4	3	3	2	2	0.49	51	80	82	80	50
13	0.42	69	4	3	3	3	2	0.44	49	80	90	80	54
14	0.14	71	4	3	3	3	2	0.39	46	90	90	90	66
15	0.21	72	4	3	3	3	2	0.36	43	90	94	90	67
16	0.07	72	4	3	3	3	2	0.34	42	90	95	90	82
17	0.28	73	5	4	3	3	2	0.31	40	90	95	90	84
18	0.42	75	5	4	4	3	3	0.19	33	90	97	90	100
19	0.14	78	5	4	5	4	3	−0.02	20	100	100	100	100
24	0.07	78	5	4	5	5	4	−0.09	2	100	100	100	100
30	0.07	78	5	5	5	5	5	−0.57	0	100	100	100	100

Abbreviations: AD = anxiety/ depression; EQ-5D-5L = 5-level version of the EuroQol 5-dimensional descriptive system; MO = mobility; PD = pain/ discomfort; PROMIS = Patient-Reported Outcomes Measurement Information System; SC = self-care; UA = usual activities; UI = utility index; US = United States; VAS = visual analog scale; WPAI:GH = Work Productivity and Activity Impairment Questionnaire: General Health

^a^The levels of 5 dimensions of EQ-5D-5L are 1 = no problems, 2 = mild problems, 3 = moderate problems, 4 = severe problems, and 5 = unable to or extreme.

^b^EQ-VAS ranges from 0 to 100. Higher values indicate better health.

^c^Utility index (United States) ranges from −0.573 to 1. Higher values indicate better health.

^d^WPAI scores range from 0 to 100. Higher values indicate more productivity loss or activity impairment.

Aligning patient-reported symptoms with validated PRO measures enabled us to propose three categories of long COVID symptom burden, defined as:

- Low symptom burden (≤2 symptoms): no problem in 3 out of 5 EQ dimensions (mobility, self-care, and usual activity); no absenteeism; fatigue within normal limits
- Medium symptom burden (3–9 symptoms): no to mild problem with mobility and self-care, mild problem with usual activity, mild to moderate problem with pain/discomfort and anxiety/depression; some impairment in work productivity and activity; mild to moderate fatigue
- High symptom burden (≥10 symptoms): at least mild problem with mobility and self-care, at least moderate problem with usual activity and pain/discomfort, severe problem with anxiety/depression; 50% or more impairment in all 4 WPAI scores; moderate to severe fatigue

Low, medium, and high symptom burden categories accounted for 75%, 21%, and 4% of analysis cohort, respectively, detailed in supplementary S4 Table.

## Discussion

Long COVID is a complex multisystem condition characterized by a plethora of symptoms that could affect patients’ wellbeing and daily activities (1–3). There is limited data evaluating the psychometric properties of symptom count-based measures and their relationships to validated PRO measures. To address this gap, this methodological study aimed to undertake a psychometric evaluation of a long COVID questionnaire derived from the prevailing literature on long COVID symptoms. The questionnaire was reviewed with medical experts to ensure comprehensive coverage of the heterogeneous manifestations of long COVID along with understandability of the symptoms listed. Results show that number of symptoms is a valid and internally consistent measure of symptom burden for various lists of symptoms.

The study methods used equipercentile linking to explore the relationship between patient-reported long COVID symptomatology and other patient-reported measures of long COVID symptom burden: HRQoL, measured with EQ-5D-5L, and work productivity and activity levels, measured with WPAI:GH. We were able to align self-reported long COVID symptoms with validated external tools to propose categories of long COVID symptom burden. Correlations between long COVID symptoms and these PRO measures showed that worsening HRQoL, impaired work productivity and activity, and greater fatigue, were all associated with a larger number of symptoms.

Moreover, regardless of which list of symptoms was used (composite 30-symptom, CDC, or HPS), a unidimensional structure was supported for long COVID symptoms, and scores expressed as number of symptoms had good internal consistency. Findings were consistent across all 3 symptom lists, and number of symptoms correlated strongly with EQ-UI (r = −0.53) and moderately with EQ-VAS (r = −0.37). Low (≤2) symptom burden had very few problems in any of the PRO measures. Medium (3–9) symptom burden had worsened HRQoL and was notable for increasing problems with pain/discomfort, anxiety/depression, and work impairment. High (≥10) symptom burden had significant problems across all PRO measures with at least 50% work impairment and notably moderate to severe pain/discomfort and fatigue, and severe anxiety/depression.

These study findings add to the limited, although growing, body of evidence evaluating data-driven groupings of long COVID symptoms. Grouping methods currently vary from study to study, either due to differences in the symptom lists used or cohort characteristics, such as severity of acute COVID-19 and sociodemographic factors [13,14,38,39]. Studies indicate that individual symptoms may vary too widely to be predictable, and not all reported symptoms may be caused by COVID-19 [40]. Such variations limit the generalizability of study findings to populations different from the study cohort.

Despite that, our study results were consistent with Ye et al., a similar study that proposed a long COVID symptom and severity score in an outpatient population. Ye et al reported a Spearman correlation coefficient of −0.55 between LC-SSS and EQ-UI and of −0.37 between LC-SSS and EQ-VAS [20]. In another study during the pandemic, the score using a list of 53 symptoms had comparable reliability (Cronbach’s α = 0.86) and Spearman correlations with EQ-UI (r=−0.46) and EQ-VAS (r=−0.39) [41].

This methodological study is subject to the limitations previously reported for the overall conduct of the study, including recall and reporting bias associated with the self-reported nature of the data [22]. Ad-hoc limitations of this methodological work should be acknowledged and discussed. First, the study did not capture symptoms prior to SARS-CoV-2 infection and whether any pre-existing symptoms worsened after SARS-CoV-2 infection. Not all symptom burden can be attributed to long COVID, i.e., these could be transient symptoms at time of completing survey, associated with other conditions. However, research shows that patients with more symptoms are more likely to have long COVID [42], and more symptoms are associated with worse PRO measures [16,21]. Further research is needed to collect pre-existing symptoms and ratings of symptoms over time, which may make it possible to assess the long COVID symptom burden more accurately and isolate the impact of long COVID on PRO measures.

Second, the study may be characterized by selection bias as the external validity may be limited to outpatients with mild illness. Patients were generally healthy, able to complete surveys by themselves, self-reported few emergency room visits (N = 11), and few were hospitalized during the study period (N = 1). This may limit the applicability of the study findings to a broader patient population, particularly older individuals and those with underlying medical conditions who may be more likely to experience severe symptoms and health outcomes associated with COVID-19. Even so, this study found that about 25% of the patient population experienced multiple persistent symptoms, acknowledging that COVID-19 symptoms burden can affect all age and risk groups and be long-lasting.

Third, the categories of scores proposed in this study were based on the linked table of PRO measures and are subject to interpretation. Although symptoms and a spectrum of PRO measures of HRQoL were used in the current study, there was no assessment of patient’s global impression of severity for the symptom score to anchor on [43]. The linked score table enables readers to explore alternative category cut‑offs or different numbers of categories.

In addition, analysis with pooled data treats repeated measures from the same patients as independent observations, which violates the independence assumption statistical methods used and inflates effective sample size. Also, the analysis cohort was predominantly female, with mild disease, in an outpatient setting. Caution should be used when applying findings to different populations, though sensitivity analyses were conducted to spot-check relationship of linked scores by repeating equipercentile linking with data from each time point or by gender separately.

Despite these limitations, this study shows that the use of a number-based symptom burden can provide a simple tool for quantifying symptom burden of long COVID for patients in the post-pandemic era. Such data might be informative, given the lack of a harmonized definition of long COVID. Moreover, the ability to classify patients in different symptom-based categories may help inform more standardized assessments and/or more personalized treatment strategies. Further longitudinal studies with longer follow-up periods, larger sample sizes and more diverse populations are warranted to continue to study long COVID symptom burden and the psychometric properties of long COVID surveys, and their relationships with PRO measures.

## Conclusion

This analysis suggests that the number of long COVID symptoms in outpatients is a valid and internally consistent measure of long COVID burden with consistent findings regardless of which long COVID symptom list was used for analysis. Aligning scores between self-reported symptoms and validated PRO measures supports data-driven groupings of long COVID symptoms. Further research is needed to develop and enhance long COVID symptom instruments in different populations and different seasons.

## Supporting information

S1 Table Lists of Long COVID Symptoms and Cronbach’s α with Deleted Variable. (PDF)

S2 Table Number of Symptoms of CDC List Linked to Patient-Reported Outcome Measures. (PDF)

S3 Table Number of Symptoms in Household Pulse Survey Linked to Patient-Reported Outcome. (PDF)

S4 Table Categories of Number of Symptoms and Linked Patient-Reported Outcome Measures. (PDF)

## Abbreviations

AD: anxiety/depression
CDC: Centers for Disease Control and Prevention
EQ-5D-5L: EuroQol 5-dimensional descriptive system
GH: General Health
HPS: Household Pulse Survey
MO: mobility
PD: pain/discomfort
PRO: patient-reported outcomes
PROMIS: The Patient-Reported Outcomes Measurement Information System
SC: self-care
SD: standard deviation
UA: usual activity
VAS: visual analog scale
WPAI: The Work Productivity and Activity Impairment Questionnaire
