# Supplementary material for: Assessment of long COVID symptom burden in patients testing positive for SARS-CoV-2 at a nationwide retail pharmacy
Source: PLoS One. 2026 Mar 25;21(3):e0345639. doi: 10.1371/journal.pone.0345639 (PMC13016359; doi:10.1371/journal.pone.0345639)
Supplement: S1 Table — (PDF) [file pone.0345639.s001.pdf]

Table S1. Lists of Long COVID Symptoms and Cronbach's α with Deleted Variable

Symptom	Cronbach's α with Deleted Variable		
	30-symptoms	CDC	HPS
With all symptoms	0.87	0.81	0.76
Tiredness or fatigue that interferes with daily life	0.86	0.79	0.73
Unusual exhaustion after exercise	0.86		0.73
Symptoms that get worse after physical or mental activities (also known as 'post-exertional malaise')	0.86	0.79	0.74
Fever	0.86	0.80	
General pain/discomfort	0.86		
Difficulty breathing or shortness of breath	0.86	0.79	0.74
Cough	0.87	0.81	
Sore throat	0.87		
Chills	0.86		
Loss of appetite	0.86		
Chest pain	0.86	0.80	0.74
Fast-beating or pounding heart (also known as heart palpitations	0.86	0.79	0.74
Difficulty thinking or concentrating (sometimes referred to as 'brain fog')	0.86	0.79	0.75
Headache	0.86	0.79	
Sleep problems	0.86	0.79	
Dizziness when you stand up (lightheadedness)	0.86	0.79	0.74
Vertigo	0.87		
Pins-and-needles feeling	0.86	0.80	
Change in smell or taste	0.87	0.81	0.76
Mood changes	0.86		
Memory loss	0.86		0.76
Confusion	0.86		
Depression or anxiety	0.86	0.79	
Diarrhea	0.86	0.80	
Stomach pain	0.86	0.79	
Nausea with or without vomiting	0.86		
Joint or muscle pain	0.86	0.79	0.74
Rash	0.87	0.81	
Hair loss	0.86		
Changes in menstrual cycles	0.86	0.80	0.73

Abbreviations: CDC = Centers for Disease Control and Prevention; HPS = Household Pulse Survey
