# Supplementary material for: Assessment of long COVID symptom burden in patients testing positive for SARS-CoV-2 at a nationwide retail pharmacy
Source: PLoS One. 2026 Mar 25;21(3):e0345639. doi: 10.1371/journal.pone.0345639 (PMC13016359; doi:10.1371/journal.pone.0345639)
Supplement: S2 Table — (PDF) [file pone.0345639.s002.pdf]

Table S2. Number of Symptoms of CDC List Linked to Patient-Reported Outcome Measures

Number of Symptoms		PROMIS Fatigue T-score	EQ-5D-5L Dimensions [a]					EQ-5D-5L Scores		WPAI: GH Scores [d]			
n	%		Anxiety / Depression	Pain / Discomfort	Usual Activities	Mobility	Self-Care	Utility Index (US) [b]	VAS [c]	Activity Impairment	Work Productivity Loss	Presenteeism	Absenteeism
0	52.3	41	1	1	1	1	1	1	93	0	0	0	0
1	18.2	51	2	1	1	1	1	0.93	85	10	10	10	0
2	9.2	54	2	2	1	1	1	0.87	80	20	30	20	0
3	6.7	57	2	2	2	1	1	0.81	76	40	47	30	4
4	4.5	60	3	2	2	2	1	0.73	71	50	55	40	21
5	2.2	62	3	2	2	2	1	0.68	70	60	63	50	50
6	2.4	64	3	3	2	2	1	0.63	65	70	70	60	50
7	1.4	65	3	3	2	2	2	0.57	60	70	77	70	50
8	1.1	66	4	3	3	2	2	0.5	54	80	80	80	50
9	0.6	68	4	3	3	3	2	0.46	50	80	90	80	53
10	0.5	71	4	3	3	3	2	0.39	46	90	90	90	66
11	0.2	73	4	3	3	3	2	0.33	41	90	95	90	83
12	0.4	74	5	4	4	3	2	0.23	39	90	97	90	100
13	0.2	78	5	4	4	4	3	0.03	22	100	100	100	100
14	0.1	78	5	4	5	5	3	-0.08	10	100	100	100	100
16	0.1	78	5	4	5	5	4	-0.09	2	100	100	100	100
19	0.1	78	5	5	5	5	5	-0.57	0	100	100	100	100

Abbreviations: EQ-5D-5L = 5-level version of the EuroQol 5-dimensional descriptive system; PROMIS = Patient-Reported Outcomes Measurement Information System; US = United States; VAS = visual analog scale; WPAI:GH = Work Productivity and Activity Impairment Questionnaire: General Health

[a] The levels of 5 dimension of EQ-5D-5L are 1=no problems, 2=mild problems, 3=moderate problems, 4=severe problems, and 5=unable to or extreme.

[b] EQ-VAS ranges from 0 to 100. Higher values indicate better health.

[c] Utility index (United States) ranges from -0.573 to 1. Higher values indicate better health.

[d] WPAI scores range from 0 to 100. Higher values indicate more productivity loss or activity impairment.
