# Supplementary material for: Assessment of long COVID symptom burden in patients testing positive for SARS-CoV-2 at a nationwide retail pharmacy
Source: PLoS One. 2026 Mar 25;21(3):e0345639. doi: 10.1371/journal.pone.0345639 (PMC13016359; doi:10.1371/journal.pone.0345639)
Supplement: S3 Table — (PDF) [file pone.0345639.s003.pdf]

Table S3. Number of Symptoms in Household Pulse Survey Linked to Patient-Reported Outcome Measures

Number of Symptoms		PROMIS Fatigue T-score	EQ-5D-5L Dimensions [a]					EQ-5D-5L Scores		WPAI: GH Scores [d]			
n	%		Anxiety / Depression	Pain / Discomfort	Usual Activities	Mobility	Self-Care	Utility Index (US) [b]	VAS [c]	Activity Impairment	Work Productivity Loss	Presenteeism	Absenteeism
0	59.3	43	1	1	1	1	1	1	92	0	0	0	0
1	16.6	52	2	2	1	1	1	0.88	82	20	20	10	0
2	9.5	57	2	2	2	1	1	0.81	78	30	40	30	0
3	5.5	59	3	2	2	2	1	0.74	71	50	55	40	20
4	3.2	63	3	2	2	2	1	0.66	69	60	65	60	50
5	2.8	65	3	3	2	2	2	0.59	62	70	73	70	50
6	1.4	67	4	3	3	2	2	0.5	51	80	80	80	50
7	0.5	70	4	3	3	3	2	0.41	49	90	90	80	56
8	0.6	72	4	3	3	3	2	0.33	41	90	95	90	82
9	0.6	78	5	4	4	4	3	0.14	28	100	100	100	100
10	0.0	78	5	4	5	5	3	-0.08	2	100	100	100	100
12	0.1	78	5	4	5	5	4	-0.09	0	100	100	100	100

Abbreviations: EQ-5D-5L = 5-level version of the EuroQol 5-dimensional descriptive system; PROMIS = Patient-Reported Outcomes Measurement Information System; US = United States; VAS = visual analog scale; WPAI:GH = Work Productivity and Activity Impairment Questionnaire: General Health

[a] The levels of 5 dimension of EQ-5D-5L are 1=no problems, 2=mild problems, 3=moderate problems, 4=severe problems, and 5=unable to or extreme.

[b] EQ-VAS ranges from 0 to 100. Higher values indicate better health.

[c] Utility index (United States) ranges from -0.573 to 1. Higher values indicate better health.

[d] WPAI scores range from 0 to 100. Higher values indicate more productivity loss or activity impairment.
