# Supplementary material for: Assessment of long COVID symptom burden in patients testing positive for SARS-CoV-2 at a nationwide retail pharmacy
Source: PLoS One. 2026 Mar 25;21(3):e0345639. doi: 10.1371/journal.pone.0345639 (PMC13016359; doi:10.1371/journal.pone.0345639)
Supplement: S4 Table — (PDF) [file pone.0345639.s004.pdf]

Table S4. Categories of Number of Symptoms and Linked Patient-Reported Outcome Measures

Number of Symptoms		PROMIS Fatigue T-score	EQ-5D-5L Dimensions [a]					EQ-5D-5L Scores		WPAI: GH Scores [d]			
Category	%		Anxiety / Depression	Pain / Discomfort	Usual Activities	Mobility	Self-Care	Utility Index (US) [b]	VAS [c]	Activity Impairment	Work Productivity Loss	Presenteeism	Absenteeism
≤2	74.7	44-53	1-2	1-2	1	1	1	0.88 - 1	>80	0-20	0-20	0-20	0
3-9	21.3	56-69	2-3	2-3	1-2	1-2	1-2	0.84 – 0.58	61 - 80	30-70	30-75	20-70	0-50
≥10	4.0	≥71	3-5	3-5	3-5	2-5	2-5	-0.57 - 0.56	≤60	>70	>75	70-100	50-100

Abbreviations: EQ-5D-5L = 5-level version of the EuroQol 5-dimensional descriptive system; PROMIS = Patient-Reported Outcomes Measurement Information System; US = United States; VAS = visual analog scale; WPAI:GH = Work Productivity and Activity Impairment Questionnaire: General Health

[a] The levels of 5 dimension of EQ-5D-5L are 1=no problems, 2=mild problems, 3=moderate problems, 4=severe problems, and 5=unable to or extreme.

[b] EQ-VAS ranges from 0 to 100. Higher values indicate better health.

[c] Utility index (United States) ranges from -0.573 to 1. Higher values indicate better health.

[d] WPAI scores range from 0 to 100. Higher values indicate more productivity loss or activity impairment.
